# Electron microscope loading and *in situ* nanoindentation of water ice at cryogenic temperatures

**DOI:** 10.1371/journal.pone.0281703

**Published:** 2023-02-10

**Authors:** Renelle Dubosq, Eric Woods, Baptiste Gault, James P. Best

**Affiliations:** 1 Max-Planck-Institüt für Eisenforschung GmbH, Düsseldorf, Germany; 2 Department of Materials, Royal School of Mines, Imperial College London, London, United Kingdom; University of Sharjah, UNITED ARAB EMIRATES

## Abstract

Interest in the technique of low temperature environmental nanoindentation has gained momentum in recent years. Low temperature indentation apparatuses can, for instance, be used for systematic measurements of the mechanical properties of ice in the laboratory, in order to accurately determine the inputs for the constitutive equations describing the rheologic behaviour of natural ice (i.e., the Glen flow law). These properties are essential to predict the movement of glaciers and ice sheets over time as a response to a changing climate. Herein, we introduce a new experimental setup and protocol for electron microscope loading and *in situ* nanoindentation of water ice. Preliminary testing on pure water ice yield elastic modulus and hardness measurements of 4.1 GPa and 176 MPa, respectively, which fall within the range of previously published values. Our approach demonstrates the potential of low temperature, *in situ*, instrumented nanoindentation of ice under controlled conditions in the SEM, opening the possibility for investigating individual structural elements and systematic studies across species and concentration of impurities to refine to constitutive equations for natural ice.

## Introduction

Glaciers and ice sheets presently cover ~10% of Earth’s land surface in alpine and polar regions, forming an integral part of the planet’s climate system, influencing regional- and global-scale climate as well as responding to climate change [1]. Our understanding of ice flow dynamics is therefore essential for forecasting glacier and ice sheet response to global warming. For instance, variations in the net mass transport of ice to the oceans can eventually lead to sea-level changes potentially drastically affecting the global water cycle [2, 3]. The dominant component of horizontal ice flow towards the oceans is shearing between the basal layer, which has a relatively higher content of chemical impurities and rock particles, and the bedrock beneath [3]. Although the rheologic behaviour of pure ice can be generalized by the Glen flow law [4], impurities introduce an enhancement coefficient as a multiplier of the stress term [5].

Based on a compilation of deformation data and mechanical tests, impurity-rich glacial ice deforms on average 2.5× faster than impurity-poor Holocene ice in simple shear [6]. While it is known that impurities affect the mechanical properties and flow behaviour of ice causing localized enhanced deformation, the effect of different impurity species at various concentrations remains ambiguous [6–8]. Therefore, simple, and systematic methods of testing the mechanical properties of ice in the laboratory while varying the species and concentration of impurities need to be developed in order to refine to constitutive equations for natural ice.

Standardized testing methods for measuring the mechanical properties of ice at millimetre length-scales currently consist of laboratory creep experiments including uniaxial compression or tension experiments, flexural testing, extrusion experiments and fracture testing [9–15]. Several groups have also applied atomic force microscopy (AFM) to measure the surface properties of ice [16–19]. The low loads available to AFM, however, limits measurements to surface forces subject to strong non-contact interactions and introduces complexities due to bending of the AFM cantilever and difficulties in the accurate determination of the tip area function.

In materials sciences, instrumented nanoindentation uses a nanometer-scale tip with known mechanical properties pressed into a material to probe its local mechanical properties [20]. Hardness and elastic modulus are then generally derived from load-displacement curves using the Oliver-Pharr analysis method [21]. Compared to macro-mechanical testing, nanoindentation has simpler specimen requirements (i.e., a flat surface), and the response of individual microstructural regions can be tested independently, enabling high throughput testing [22, 23]. Advances in instrumented nanoindentation also allow for testing of micro-geometries, including micro-cantilevers for fracture toughness or micro-pillars for strength measurements for instance [24].

The last few decades have seen significant developments in low temperature nanoindentation. In one type of apparatus, the specimen and indenter can be fully immersed in a cryogenic liquid contained in an insulated vessel [25–27]. For such systems, the testing temperature is limited to the natural boiling point of liquid nitrogen (LN_2_, 77 K) or liquid helium (LHe, 4.2 K). Temperature control is challenging, and the constant formation of gas bubbles in the liquid cells result in turbulence that affect the load measurements during indentation. In another type of apparatus, the indenter can be retrofitted with refrigeration systems (e.g., Gifford-McMahon refrigerator, Peltier coolers) and electric heaters to control the temperature of the specimen and indenter independently [22, 28–30]. To prevent frost contamination, newer setups operate inside a scanning electron microscope (SEM) which allow for *in situ* testing and observations under vacuum, together with precise control over the tip, sample and frame temperatures for minimised thermal drift.

Herein, we introduce an experimental protocol to conduct *in situ* instrumented nanoindentation of water ice using an Alemnis Low Temperature Module (LTM-CRYO) installed within a SEM. While the LMT-CRYO is a commercial product, we demonstrate a new procedure for preparing and loading ice onto the nanoindentation stage, cooling, testing and venting while preserving the specimen surface. We detail the setup and steps necessary to obtain preliminary measurements on the elastic modulus (*E*) and hardness (*H*) for pure water ice and offer key insights into how these measurements could be further optimised by researchers. Our approaches pave the way for complex nano- and micromechanical testing of ice and its individual structural elements, along with the possibility of high throughput, under controlled conditions in the SEM.

## Materials and methods

### Hardware

**Fig 1:**
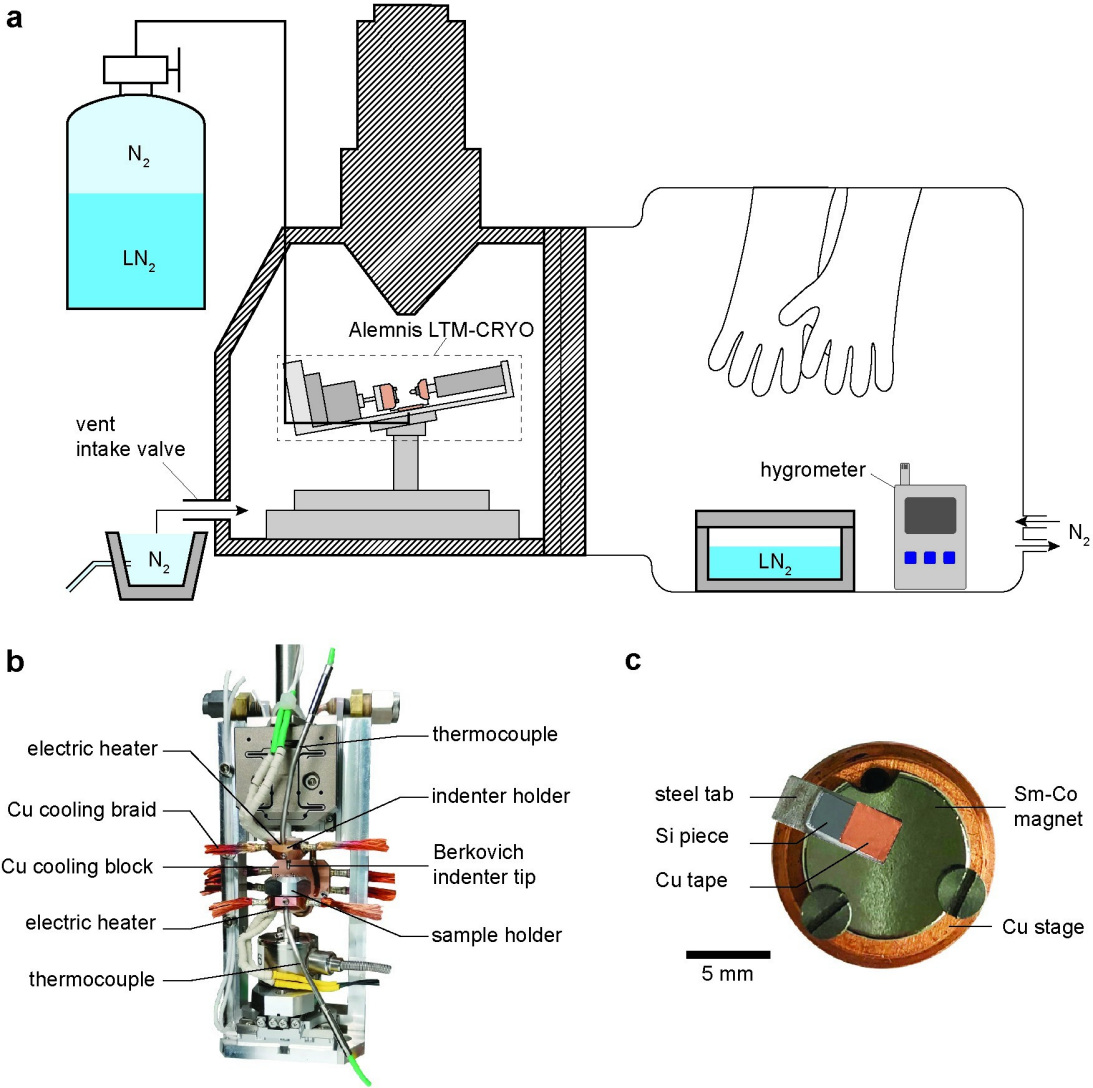
Schematic diagram of the experimental setup for in situ instrumented nanoindentation experiments of water ice (a). Photographs of the Alemnis LTM-CRYO indentation device (supplied by Alemnis AG) (b) and sample holder (c).

The experimental setup for *in situ* instrumented nanoindentation of water ice in a controlled low temperature environment is outlined in Fig 1A. We used an Alemnis LTM-CRYO (Alemnis AG, Switzerland) retrofitted to an Alemnis Standard Assembly (ASA) equipped with SEM feedthroughs for power and temperatures control. The system has load and displacement resolutions of 4 μN and <1 nm, respectively. This system was mounted into a JEOL JSM-6490 instrument (JEOL Instruments, Tokyo, Japan) for *in situ* observations.

To avoid frost contamination on the ice samples and on the indentation device during loading, we created an oxygen depleted atmosphere by attaching a glovebag to the outside of the SEM chamber and flowing in nitrogen gas (N_2_). We used an Aldrich® AtmosBag two-hand, non-sterile, size L, with zipper-lock (Sigma Aldrich, St. Louis, MO, USA). During the experiments, the glovebag is completely sealed therefore all the needed tools and materials must be placed inside the bag prior to sealing (i.e., LN_2_-filled dewar, water flask, pipette, tweezers, sample tablets and hygrometer).

The LTM-CRYO (Fig 1B) comprises a sample holder and an indenter holder with a conductive diamond Berkovich indenter, both with separate temperature control loops and cooling capability down to -150°C under vacuum conditions. Cooling is achieved via LN_2_ flow through a Cu cooling block and Cu braids and regulated by electric heaters and thermocouples using a temperature control software. To facilitate sample insertion, the sample holder was adapted by placing a circular Sm-Co magnet (10 mm diameter × 2 mm thickness, First4Magnets, China) with circular cut-outs (3 mm diameter) along the edges at 120° angles to allow space for the sample holder screws (Fig 1C).

In addition, small sample tablets were designed onto which the ice samples can be prepared (Fig 1C). The tablets consist of a polished Si piece (7 × 3 × 0.5 mm) cut from 50 mm diameter test grade <100> single crystal Si wafers, glued onto a rectangular steel tab (10 × 4 × 1 mm; stainless steel 304) using an epoxy with high shear strength at cryogenic temperatures (i.e., EPO-TEK T7110, Epoxy Technology Inc., Billerica, MA, USA; Fig 1C). A piece of Cu tape (Plano GmbH, Wetzlar, Germany) was placed on top of the Si piece to increase the surface wettability and ensure stability during sample transfer. The steel tab protrudes from the magnet edge, which acts as a "lip" allowing for manipulation with the use of tweezers, while the magnet facilitates rapid sample loading into the SEM chamber during experiments.

### Experimental protocol

To begin the experiments, the SEM chamber is vented and the door opened. After attaching the glove bag to the outside of the SEM chamber, N_2_ flow (≥99.999% purity) from an N_2_ line to the glovebag is initiated until the lowest possible dew point is reached. With this setup, a dew point of -35°C was achieved after approximately 30 minutes of purging. At this stage, the LTM-CRYO indentation system is cooled, including sample and indenter holders, to below 0°C but still above the dew point.

**Fig 2:**
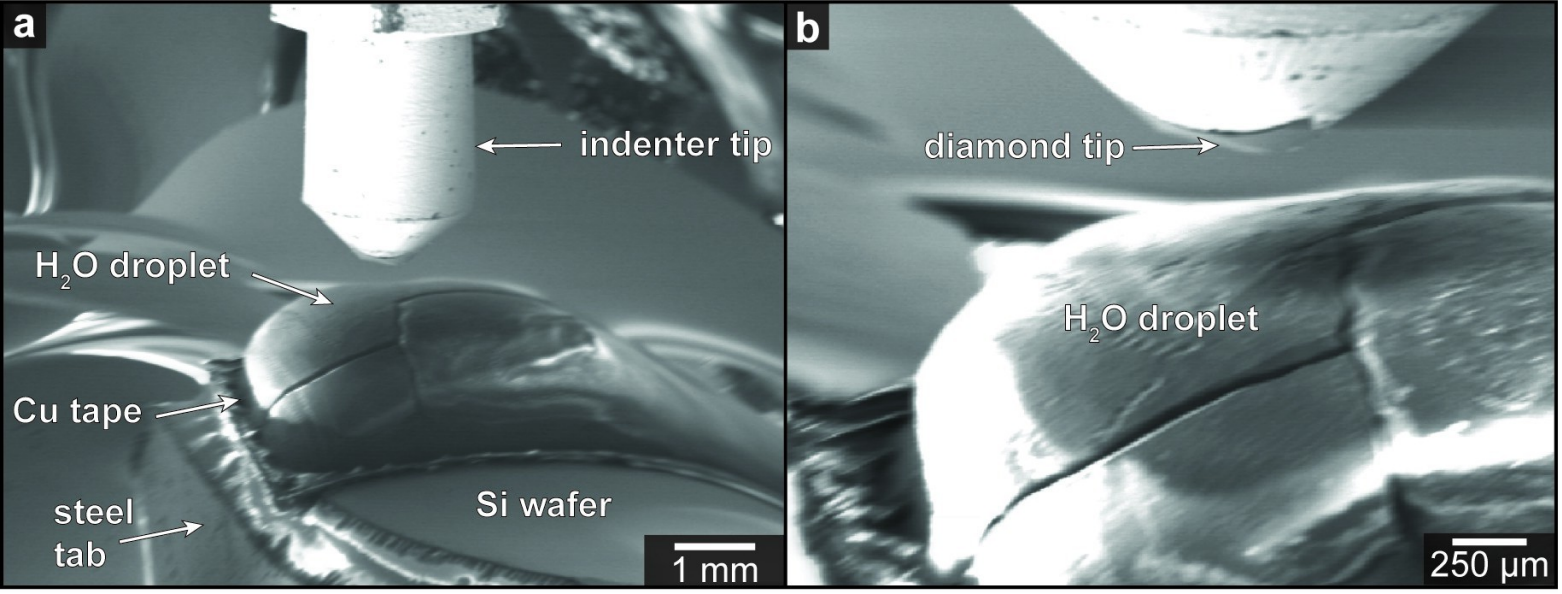
(a, b) SE images of water ice sample and indenter tip prior to experiments. Note image distortion generated by the electromagnetic interference of the Sm-Co magnet.

In parallel, the ice sample is prepared by placing a water droplet onto the Cu tape on the tablet and submerging it into LN_2_. For the purpose of this study, we used type 1 (18 MΩ) deionized water for preparing our ice samples. Once the sample holder on the LTM-CRYO reaches a temperature of ~ -20°C, the ice sample is placed onto the holder by snapping the steel tablet into place on the magnet, with the ice droplet placed as close as possible to the holder center. Sample loading must be performed before the sample and indenter holders reach the dew point temperatures in order to avoid frost contamination. Before closing the SEM door, the indenter tip was aligned with the sample. After evacuating the chamber, the sample and indenter holder temperatures can be stabilized by regulating the LN_2_ flow to the indentation system and controlling the power on the electric heaters to the desired testing temperature. Secondary electron images of the ice sample prior to indentation are shown in Fig 2.

Upon completion of the indentation experiments, the N_2_ gas flow to the glovebag is once again initiated to prepare for sample unloading. Once the atmosphere stabilizes at the minimum dew point (~ -35°C), the LN_2_ flow to the sample and indenter holders is stopped to slowly warm up the indentation system, up to a temperature slightly above the dew point (> -35°C) of the glovebag atmosphere yet still at freezing conditions. Since the JEOL JSM-6490 vents using ambient air, we place a container connected to a direct N_2_ line near the intake valve to ensure a direct feed-in of N2 gas at atmospheric pressure into the SEM chamber to reduce the humidity during the venting procedure and prevent frost formation (Fig 1A). After venting the chamber, the ice sample is removed from the holder and submerged in LN_2_ for preservation.

## Results and discussion

### Elastic modulus and hardness measurements

**Fig 3:**
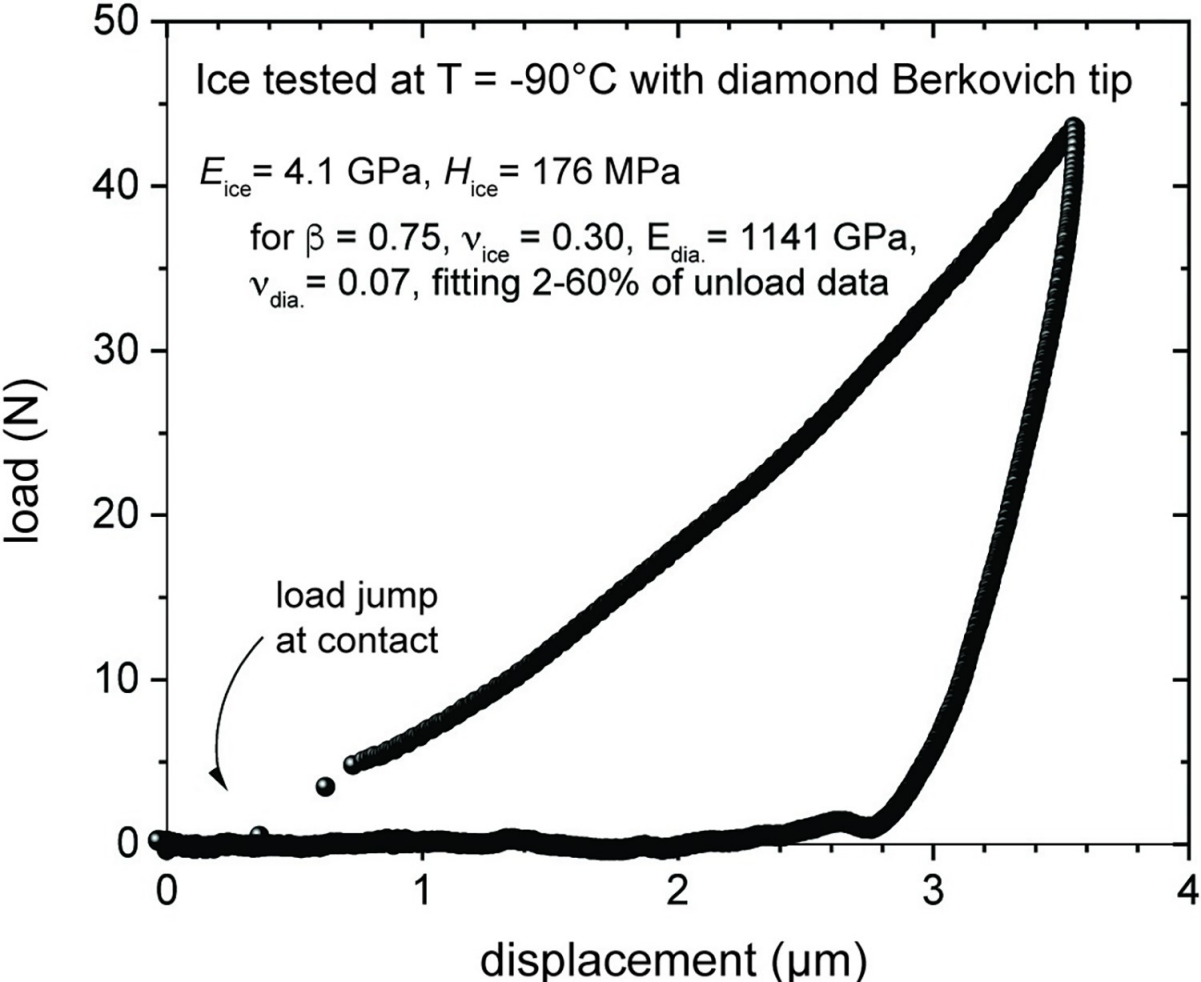
Representative load versus indenter displacement curve of experiments performed on water ice sample with diamond Berkovich nanoindentation tip.

Nanoindentation was performed on one sample at a constant loading rate until reaching a peak load of 44 μN before unloading without a hold segment in order to minimize the effect of sublimation or melting of the ice surface. The loading and unloading experiments were conducted at a constant temperature of -90°C. A total of four indents were made, however, due to various complexities during the experiment, we report one single load-displacement curve to demonstrate the potential of our experimental setup and protocol (Fig 3; S1 File). The Young’s modulus (*E*) and hardness (*H*) for the experiment was calculated from the load-displacement curve following the Oliver-Pharr analysis method [21]. The geometrical correction factor β of 0.75 and a Poisson’s ratio (ν) of 0.30 for ice, and an *E* of 1141 GPa and ν of 0.07 for the diamond Berkovich indenter were used for the calculations and the curve was optimized to fit 2–60% of the unload data. The maximum indentation depth was 3.56 μm yielding a *E* of 4.1 GPa and a *H* of 176 MPa for the ice sample. *E* agrees reasonably with previous measurements from laboratory experiments (*E* ~9–11 GPa) [31–33] and from field observations (*E* ~1 GPa) [34–36]. Discrepancies between laboratory and field measurements have been attributed to variations in the loading rates and the magnitude of applied stresses. Measurements using field techniques rely on observing the response of ice shelves to tidal deformation which corresponds to low loading frequencies and high stresses. Under such conditions, ice may fracture and creep instead of deforming elastically, which can lead to erroneous calculations for *E* [34, 37]. Based on this theory, the proximity of the indents to fractures in our ice sample (Fig 2) could potentially account for the disparity between our *E* measurements and those reported from laboratory experiments. To avoid fractures, future ice samples could be slowly cooled by using an apparatus that allows for the precise control of the freezing kinetics [38].

In AFM studies, it has been demonstrated that the pressure exerted by the tip can either lead to the plastification or the interfacial pre-melting of the ice surface [39–44] once again leading to the underestimation of *E*. Although nanoindentation should not be as sensitive to surface interactions due to the much higher loads used for testing, it is still a surface sensitive technique therefore results could still be affected by plastification or interfacial pre-melting. Other factors that could contribute to underestimating *E* include the roughness and curvature of the frozen droplet surface. When indenting a curved surface, the asymmetry of the indent can introduce inaccuracies to the simple functional relationship used for estimating the contact area (*A*). The surface curvature of the ice sample could be minimized by substituting the Si wafer substrate with e.g. a porous material to increase the wettability of the surface (e.g., oxidized Si wafer, nanoporous gold) [45]. While choosing a substrate, however, one must also consider the potential effects of its mechanical properties on the determination of the micromechanical measurements of the ice layer.

Although studies on ice hardness are currently very limited, our *H* measurements also fall within the range of previously published measurements for water ice. For example, Pittenger et al. [46] measured ice hardness by indenting the surface of ice at temperatures of -1– -15°C using sharp AFM tips and yielded maximum *H* values in the range of 10–300 MPa. These values, however, were significantly higher than those measured by previous macroscopic indentation techniques on polycrystalline ice which measured *H* values of 10–50 MPa at temperatures of 0– -15°C [47, 48]. In these studies, the softer measurements are believed to be due to the viscous flow of a quasiliquid layer at the ice surface as a result of interfacial pre-melting. The slight disparity between our measurements and those of previous studies could be due to the temperatures used for our experiments. Our measurements were conducted at significantly cooler temperatures, at which the hardness is expected to be higher. Additionally, since $E\propto S/\sqrt{A}$ and *H*∝*P/A*, where *S* is the measured stiffness and *P* is the peak load, *H* measurements are more sensitive to errors in the estimation of the contact area [21], which may explain the large range of published *H* measurements. The significant variability between the reported values for *E* and *H* demonstrate the impetus to develop new methodologies for performing more systematic testing using established and robust techniques (i.e., nanoindentation). Such techniques allow for a large number of experiments to be conducted at various conditions to deepen our understanding of ice mechanics.

### Challenges and future developments

Although we have successfully performed the first *in situ* instrumented nanoindentation of water ice within an SEM, yielding reasonable *E* and *H* measurements, further technical developments are required to make it routine and combine it within correlative analytical microscopy workflows to link structure-composition and properties. Such approaches require that the sample surface is stable, and the features created during the nanoindentation experiments be maintained for the duration of the analysis in the SEM, for e.g. electron backscatter diffraction (EBSD). The stability of ice is dependent on the pressure and temperature conditions in the SEM chamber, as these parameters control the thermodynamics and kinetics of sublimation and condensation, making the transfer between instruments critical to enable possible further analyses by cryogenic-transmission electron microscopy or cryo-atom probe tomography following preparation by focused-ion beam milling at cryogenic temperature [49–52].

**Fig 4:**
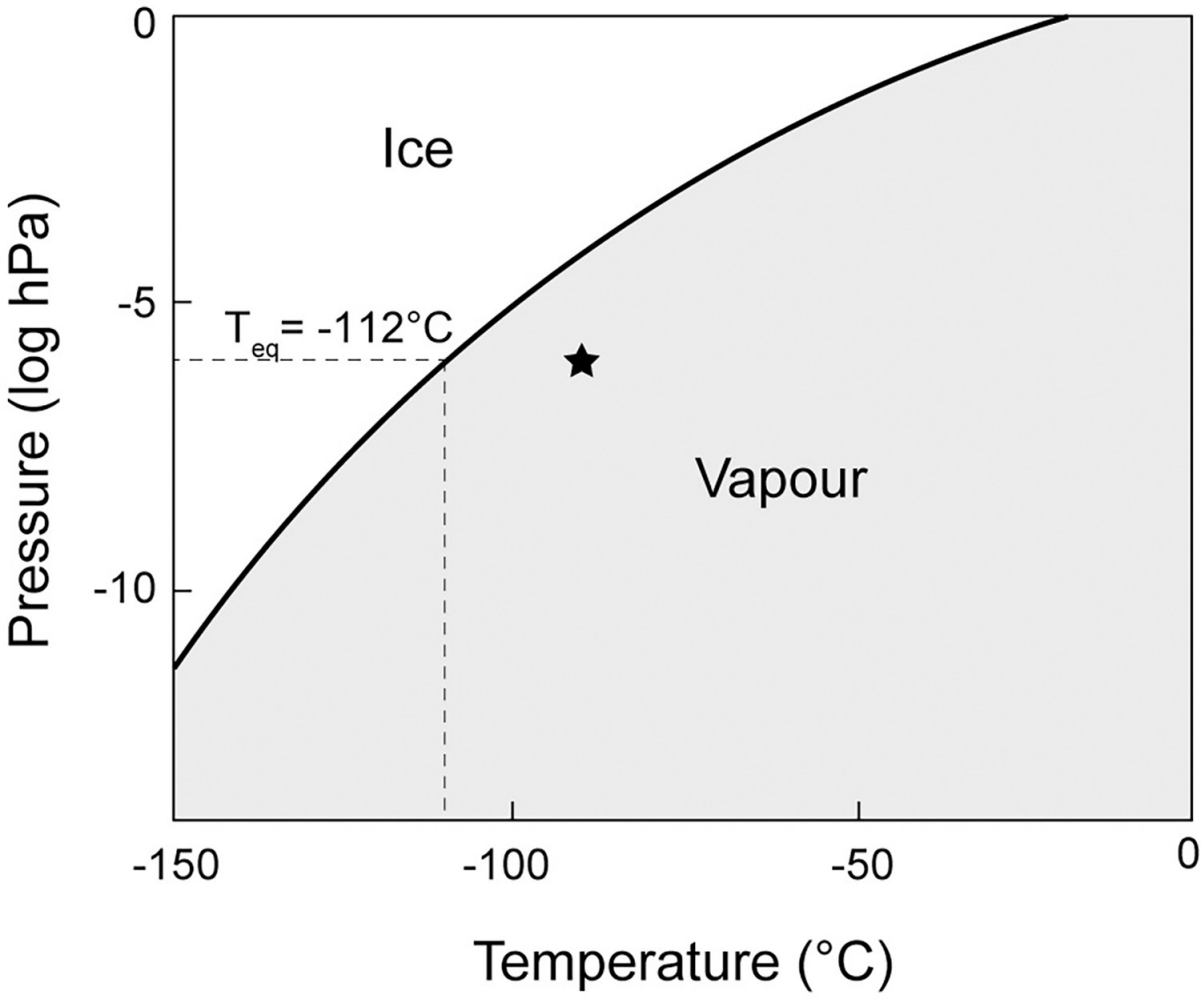
Equilibrium phase diagram showing stability conditions for water ice and vapour in a closed system (modified after Andreas, 2007 and Weikusat et al., 2011). The equilibrium temperature for a chamber pressure of 1×10^−6^ hPa is approximately -112°C. The SEM chamber pressure and temperature for the current study’s experiments are shown with a star.

Under high vacuum SEM conditions, the chamber pressure is typically on the order of 1×10^−6^ hPa. At this pressure, the equilibrium temperature occurs at approximately -112°C. At equilibrium, as indicated in Fig 4, the condensation rate equals the sublimation rate and the sample surface can be preserved [53, 54]. Herein, our experiments were conducted at temperatures of -90°C, i.e. in conditions under which ice is unstable and sublimates. Based on calculations from previous studies, the sublimation rate of ice at -90°C and 1×10^−6^ hPa is estimated to be approximately 1 μm min^-1^ [55–57]. At this rate, the indents created during our experiments are quickly lost and it is no longer possible to correlate these features with other characterization techniques. Such high sublimation rates could also lead to inaccuracies in contact depth calculations and signal drifts, potentially leading to errors in *E* and *H* calculations. Nanoindentation experiments on water ice at the tested vacuum pressure should therefore be performed below temperatures of -112°C for correlative studies, or alternatively using SEMs with the possibility for low vacuum mode.

Additional limitations of the current approach include temperature variations during sample preparation and loading and unloading procedures. In this study, the samples were prepared by submerging a droplet of water into LN_2_. Therefore, the ice is quickly cooled to -195.8°C. After freezing, since the current set up can only reach a dew point of ~ -35°C, sample loading onto the indentation device must occur at slightly higher temperatures to avoid frost contamination. During the nanoindentation experiments, the ice sample can be cooled to the desired temperature, however, it must be warmed again to temperatures above -35°C for unloading. Since ice crystal structure varies with temperature [58], such variations need to be minimized to avoid phase changes during sample preparation and transfer procedures for correlative analyses.

These issues can be alleviated by using a new glovebox with a load lock and port for a high-vacuum cryo transfer system that can be attached to the SEM. A glovebox can be constantly purged with dry N_2_ gas keeping the humidity at a minimum. To avoid introducing humidity during sample unloading, the SEM also needs to be adapted to vent using N_2_. With this setup, the nanoindentation device could be cooled to lower temperatures while still preventing frost contamination and the temperature variations between the ice sample and the sample holder would be minimized. The addition of a port for a high vacuum cryo transfer system [59] would facilitate sample handling between instruments for correlative analysis.

Lastly, although one of the advantages of conducting *in situ* nanoindentation within an SEM is the ability to visualize the experiments and capture images of the sample surface prior and post indents, the resolution and the quality of the images are currently limited by the distortions generated by the electromagnetic interferences of the Sm-Co magnet on the sample holder (Fig 2). The use of a magnetic holder is critical to ensure rapid transfer of the sample in our current set-up and minimise thermal losses. Therefore, to mitigate this issue we propose the use of a weaker magnet that maintains its magnetism under cryogenic conditions (e.g., Al-Ni-Co).

## Conclusion

In this study, we have designed a simple experimental setup and protocol that allowed for the first *in situ* instrumented nanoindentation of water ice in a controlled low temperature environment using an Alemnis LTM-CRYO installed within a SEM. Preliminary nanoindentation experiments on pure water ice yield *E* and *H* measurements of 4.1 GPa and 176 MPa, respectively, which are in reasonable agreement with previously published values. The experimental protocol presented in this study paves the way for micro- to nanomechanical measurements of microstructural features in ice where the chemistry and structures (e.g., grain size) can be varied. However, as outlined, future technical developments are necessary to optimize this approach and link low temperature nanoindentation experiments to various correlative microscopy techniques.

## Supporting information

S1 File Optimised and corrected nanoindentation data on ice. (XLSX)
