# Correction: Electron microscope loading and in situ nanoindentation of water ice at cryogenic temperatures

**DOI:** 10.1371/journal.pone.0306374

**Published:** 2024-06-27

**Authors:** Renelle Dubosq, Eric Woods, Baptiste Gault, James P. Best

In the Elastic modulus and hardness measurements subsection of Results and Discussion, there is an error in the first sentence of the first paragraph. The correct sentence is: Nanoindentation was performed on one sample at a constant loading rate until reaching a peak load of 44 mN before unloading without a hold segment in order to minimize the effect of sublimation or melting of the ice surface.

Additionally, Fig 3 is incorrect. The authors have provided a corrected version here.

**Fig 3:**
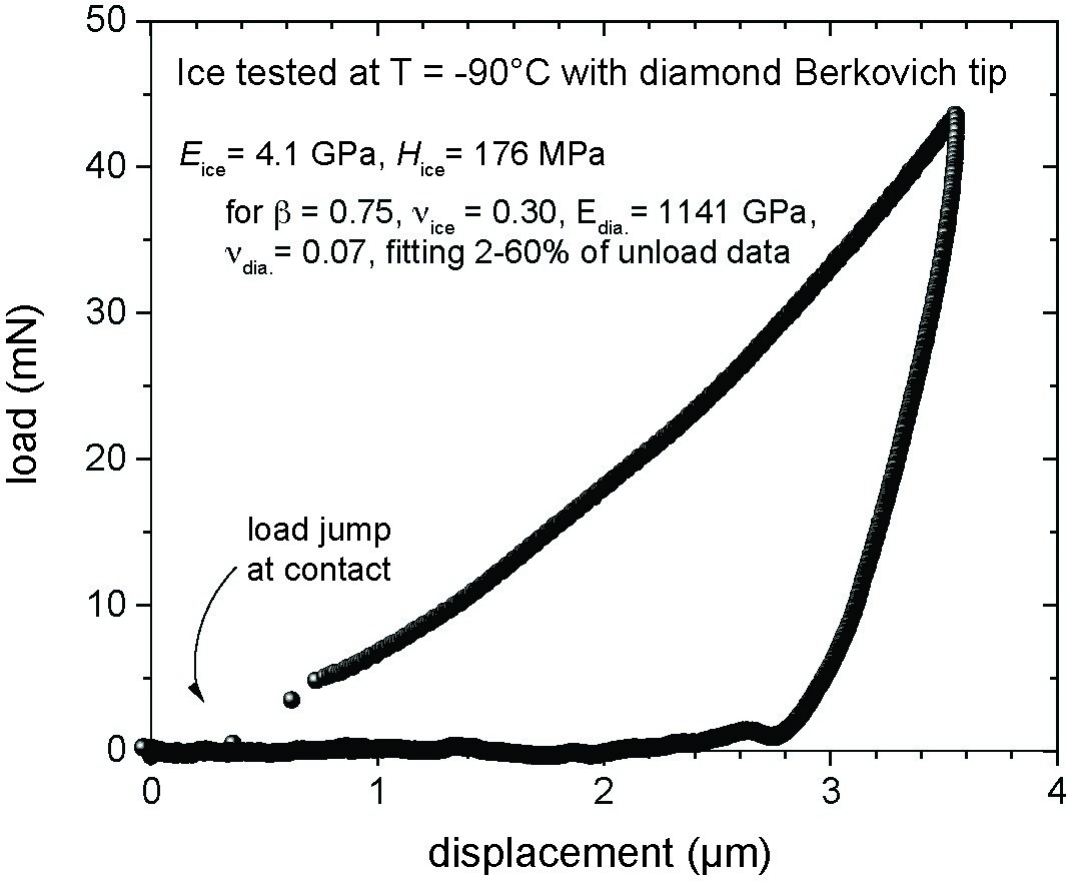
Representative load versus indenter displacement curve of experiments performed on water ice sample with diamond Berkovich nanoindentation tip.
